# Novel agents and regimens in relapsed or refractory peripheral T-cell lymphoma: latest updates from 2023 ASH annual meeting

**DOI:** 10.1186/s40164-024-00510-w

**Published:** 2024-04-22

**Authors:** Huageng Huang, Wei Zhang, Xinyi Deng, He Huang, Zhao Wang, Huangming Hong, Tongyu Lin

**Affiliations:** 1 Department of Medical Oncology, State Key Laboratory of Oncology in South China, Guangdong Key Laboratory of Nasopharyngeal Carcinoma Diagnosis and Therapy, Guangdong Provincial Clinical Research Center for Cancer, Sun Yat-sen University Cancer Center, Guangzhou, 510060 China; 2 Department of Medical Oncology, Senior Ward and Phase I Clinical Trial Ward, Sichuan Cancer Center, School of Medicine, Sichuan Cancer Hospital & Institute, University of Electronic Science and Technology of China, Chengdu, 610000 Sichuan China; 3 Department of Dermatology, The First Affiliated Hospital of Guangzhou Medical University, Guangzhou, 510120 Guangdong China

**Keywords:** Peripheral T-cell lymphoma, Relapse, Refractory, Clinical research

## Abstract

Peripheral T-cell lymphoma (PTCL) is a heterogeneous group of hematological malignancies with poor survival, while treatment options for relapsed or refractory (R/R) disease remain quite limited, with a median progression-free survival of only 3–4 months. Notably, the emergence of innovative therapeutic agents and regimens holds promise for durable responses and improved survival for patients with R/R PTCL. We summarize recent advances in the treatment of R/R PTCL from the 2023 ASH Annual Meeting, highlighting novel agents targeting EZH1/2, JAK1, PI3K, KIR3DL2, CD38/CD3xCD28, or CDK9, as well as therapeutic regimens in combination with stem cell transplantation, immunomodulators, epigenetic modifying agents, or CD30/CD16A bispecific antibodies.

## To the editor

Peripheral T-cell lymphoma (PTCL) comprises a heterogeneous group of hematological malignancies where relapsed or refractory (R/R) disease is common. Current standard treatments for R/R PTCL have limited efficacy, and new therapeutic methods are urgently needed [1, 2]. We summarize the latest updates on novel agents and regimens for R/R PTCL from the 2023 ASH Annual Meeting.

## Novel agents in R/R PTCL

Valemetostat, a potent dual inhibitor of Enhancers of Zeste Homolog 1 and 2 (EZH1/2), is approved for the treatment of R/R adult T-cell leukemia/lymphoma in Japan. In a multinational pivotal phase II trial of R/R PTCL (VALENTINE-PTCL01), continuous administration of 200 mg/day valemetostat was well tolerated and displayed an objective response rate (ORR) of 43.7% (52/119) with 14.3% complete response (CR) and a median duration of response (DOR) of 11.9 months. Responses were observed across all PTCL subtypes. The median progression-free survival (PFS) and overall survival (OS) were 5.5 months and 17.0 months, respectively [3]. Another exciting EZH1/2 dual inhibitor HH2853 was evaluated in a phase Ib trial. Among 28 R/R PTCL patients with assessable efficacy, HH2853 resulted in a 60.7% ORR with 21.4% CR. With a median follow-up duration of 2.79 months, the median DOR, PFS and OS were not achieved. The 3-month PFS rate and 6-month OS rate were 74.44% and 91.97%, respectively. Trentment-related adverse events (TRAEs) were manageable and consistent with other HH2853 studies [4].

Golidocitinib is the first JAK1 selective inhibitor to enter pivotal clinical development for R/R PTCL. In the global phase II trial (JACKPOT8 Part B), patients with R/R PTCL were given 150 mg/day of golidocitinib continuously and showed a 44.3% (39/88) ORR, including 24% CR, with a median DOR, PFS and OS of 20.7 months, 5.6 months and 19.4 months, respectively. The majority of treatment-emergent adverse events (TEAEs) were reversible and clinically manageable [5].

Linperlisib, a novel PI3Kδ-selective inhibitor, has received marketing approval in China for the treatment of R/R follicular lymphoma patients with 2 prior systemic therapies. In a pivotal phase II study of R/R PTCL, patients were consecutively administered linperlisib 80 mg/day, achieving 48% (42/88) ORR with 30% CR and median PFS and OS of 5.5 months and 14.2 months, respectively. Activity was demonstrated in all subtypes, and the median DOR was not reached at a median follow-up of 13.9 months. Linperlisib has a favorable safety profile, with lower levels of severe gastrointestinal and liver toxicity than other PI3K agents [6]. BR101801, a triple inhibitor of PI3Kγ/δ and DNA-PK, showed clinical benefit and a manageable safety profile in a phase I trial of R/R PTCL: 31.6% (6/19) ORR, 21.1% CR, and a median PFS of 7.5 months; median OS and DOR were not reached in a median follow-up duration of 12.9 months; grade 3–4 TRAEs included elevated transaminases and neutropenia [7].

Preliminary phase Ib data on the anti-KIR3DL2 mAb lacutamab in 10 patients with KIR3DL2-expressing R/R PTCL confirmed an acceptable safety profile for monotherapy, with the majority (90%) of TEAEs being of grade 1–2 severity [8]. The preclinical activity profile improved when assessed in combination with pralatrexate or CHOP. Two additional preclinical studies revealed superior antitumor activity against PTCL with the CD38/CD3xCD28 trispecific antibody SAR442257 monotherapy [9] and the CDK9 inhibitor AZD4573 monotherapy or in combination with CHOP [10].

## Novel regimens in R/R PTCL

A multicenter retrospective study of 184 patients with R/R PTCL revealed that novel agent exposure with and without the use of stem cell transplantation (SCT) significantly improved OS and event-free survival [11]. For patients who respond after salvage therapy but are ineligible for autologous SCT or intensive consolidation chemotherapy, a multicenter phase II trial demonstrated that maintenance therapy with the immunomodulator lenalidomide 50 mg for 3 weeks in 4-week cycles was tolerable and produced promising responses, with a 1-year PFS of 49%, a median OS of 34.1 months and manageable TRAEs [12].

The histone deacetylase inhibitor chidamide combined with the hypomethylating agent azacitidine for injection was evaluated in a phase I trial for R/R PTCL. This pair was well tolerated and resulted in an ORR of 56.25% (9/16), with a median PFS and OS of 6.45 months and 17.5 months, respectively [13]. Chidamide has also been studied in combination with parsaclisib, a potent PI3Kδ-selective inhibitor, in a phase Ib/II trial of R/R PTCL. Preliminary results showed a favorable safety profile, with predominantly grade 1/2 hematologic toxicities and gastrointestinal reactions. Among the 9 efficacy evaluable patients, 5 (55.6%) achieved CR, and patients previously exposed to chidamide could still have durable responses [14].

An ongoing phase II study (LuminICE) was designed to explore the efficacy and safety of the CD30/CD16A bispecific antibody (AFM13) in combination with allogeneic natural killer cells (AB-101) in R/R Hodgkin lymphoma and CD30 + PTCL [15].

In conclusion, the 2023 ASH Annual Meeting showcased many notable advances in new agents and novel rational drug combinations targeting the epigenome, proliferative signaling pathways, tumor microenvironment and cell surface receptors for the treatment of R/R PTCL, as summarized in Tables 1 and 2. A deeper understanding of the molecular and genomic profiles will provide potential therapeutic targets for PTCL. Notably, with technological innovations, chimeric antigen receptor (CAR)-T cells targeting CD5, CD7, CD30, CD70 or TRBC1 have been developed and are being explored in clinical trials in selected patients with R/R PTCL.

**Table 1:** Outcomes of clinical trials of novel agents in relapsed or refractory PTCL from 2023 ASH Annual Meeting

Agent	Target	Phase	Accrual	Major subtype	Median prior therapy	Response	Survival	Major grade 3–4 AEs	Abstract number	References
Valemetostat	EZH1/2	II	133	AITL (31.6%), PTCL-NOS (30.8%), ALK-negative ALCL (5.3%), ALK-positive ALCL (1.5%), PTCL-TFH (6.0%), other (14.3%)	2	ORR 43.7%, CR 14.3%, PR 29.4%, mDOR 11.9 m	mPFS 5.5 m, mOS 17.0 m	Thrombocytopenia (23.3%), Anemia (18.8%), Neutropenia (17.3%)	302	[3]
HH2853	EZH1/2	Ib	34	AITL (41.2%), PTCL-NOS (32.4%), ALK-negative ALCL (11.8%), NKTCL (5.9%), PTCL-TFH (5.9%), SKIN-PTCL (2.9%)	2	ORR 60.7%, CR 21.4%, PR 39.3%, SD 14.3%, mDOR not reached	mPFS not reached, 3-m PFS rate 74.44%, mOS not reached, 6-m OS rate 91.97%	Thrombocytopenia (14.7%), Neutrophil count decreased (11.8%)	304	[4]
Golidocitinib	JAK1	II	104	PTCL-NOS (57%), AITL (18%), ALCL (11%), NKTCL (3%)	2	ORR 44.3%, CR 24%, PR 20%, SD 19%, mDOR 20.7 m	mPFS 5.6 m, mOS 19.4 m	Neutrophil count decreased (29%), White blood cell count decreased (26%), Lymphocyte count decreased (21%), Thrombocytopenia (20%)	305	[5]
Linperlisib	PI3Kδ	II	98	AITL (49%), PTCL-NOS (24%), NKTCL (8%), ALCL (2%)	2	ORR 48%, CR 30%, PR 18%, SD 20%, mDOR not reached	mPFS 5.5 m, mOS 14.2 m	Neutropenia (32%), Pneumonia (14%), Leukopenia (10%), Anemia (6%), Thrombocytopenia (5%), Upper respiratory tract infection (5%), Lymphocytopenia (5%)	306	[6]
BR101801	PI3K γ/δ and DNA-PK	Ib	26	PTCL-NOS (42%), AITL (42%)	2	ORR 31.6%, CR 21.1%, PR 10.5%, mDOR not reached	mPFS 7.5 m, mOS not reached	AST increased (19.2%), ALT increased (15.4%), Neutropenia (15.4%)	1701	[7]
Lacutamab	KIR3DL2	Ib	10	N/A	3	N/A	N/A	Serum sickness (10%), AST increased (10%)	3072	[8]

Abbreviations: PTCL, peripheral T-cell lymphoma; ASH, American Society of Hematology; AE, adverse event; EZH1/2, Enhancers of Zeste Homolog 1 and 2; JAK1, Janus kinase 1; PI3K, phosphatidylinositol 3-kinase; AITL, angioimmunoblastic T-cell lymphoma; PTCL-NOS, PTCL, not otherwise specified; ALK, anaplastic lymphoma kinase; ALCL, anaplastic large cell lymphoma; PTCL-TFH, nodal PTCL with T follicular helper cell phenotype; NKTCL, natural killer/T cell lymphoma; N/A, not available; ORR, objective response rate; CR, complete response; PR, partial response; SD, stable disease; mDOR, median duration of response; mPFS, median progression-free survival; mOS, median overall survival; AST, aspartate aminotransferase; ALT, alanine aminotransferase

**Table 2:** Outcomes of novel regimens in relapsed or refractory PTCL from 2023 ASH Annual Meeting

Regimen	Study type	Accrual	Major subtype	Median prior therapy	Efficacy	Major AEs	Abstract number	References
Novel agent + SCT vs. Chemotherapy + SCT vs. Novel agent vs. Chemotherapy	Retrospective	184	AITL (28.3%), PTCL-NOS (21.2%), ATLL (8.5%), and ALCL (13%)	N/A	mEFS 16.0 m vs. 15.0 m vs.3.9 m vs. 4.1 m, mOS 70.7 m vs. not reached vs. 23.3 m vs. 16.7 m	N/A	1698	[11]
Lenalidomide maintenance after salvage therapy	Phase II	58	AITL (39.7%), PTCL-NOS (25.9%)	N/A	1-y PFS 49%, mOS 34.1 m	Hematologic toxicities: Neutropenia, Thrombocytopenia; Non-hematologic toxicities: Skin rash (40%), Nausea (35%), Diarrhea (20%)	3073	[12]
Chidamide + Azacitidine	Phase I	19	AITL (57.9%), PTCL-NOS (42.1%)	2	ORR 56.25%, DCR 75%, mPFS 6.45 m, mOS 17.5 m	Pain at the injection site (75.0%), Neutropenia (56.3%), Erythema at the injection site (50.0%), Nausea (43.8%), Thrombocytopenia (37.5%), Feeble (37.5%), Anemia (31.3%), Diarrhea (31.3%), Neutropenia with fever (25.0%)	3076	[13]
Chidamide + Parsaclisib	Phase Ib/II	11	N/A	2	CR 55.6%	Neutropenia (36.4%), Leukopenia (9.1%), Elevation of ALT/AST (9.1%), Diarrhea (9.1%)	3075	[14]

Abbreviations: PTCL, peripheral T-cell lymphoma; ASH, American Society of Hematology; AE, adverse event; AITL, angioimmunoblastic T-cell lymphoma; PTCL-NOS, PTCL, not otherwise specified; ATLL, adult T-cell leukemia/lymphoma; ALCL, anaplastic large cell lymphoma; mEFS, median event-free survival; PFS, progression-free survival; mOS, median overall survival; ORR, objective response rate; DCR, disease control rate; CR, complete response; N/A, not available; AST, aspartate aminotransferase; ALT, alanine aminotransferase

## Data Availability

The material supporting the conclusion of this study has been included in the article.

## Abbreviations

CAR: Chimeric antigen receptor
CR: Complete response
DOR: Duration of response
EZH1/2: Enhancers of Zeste Homolog 1 and 2
JAK1: Janus kinase 1
ORR: Objective response rate
OS: Overall survival
PFS: Progression-free survival
PI3K: Phosphatidylinositol 3-kinase
PTCL: Peripheral T-cell lymphoma
R/R: Relapsed or refractory
SCT: Stem cell transplantation
TEAE: Treatment-emergent adverse event
TRAE: Treatment-related adverse event
